# Meta-path guided policy distillation for resilient coordination in autonomous unmanned swarm

**DOI:** 10.1371/journal.pone.0339675

**Published:** 2025-12-31

**Authors:** Xingye Han, Huifang Wang, Qiang Jia, YingDong Gou, Bo Li, Jiancheng Liu, Zaikun Han, Gang Hou, Ke Li, Junxiong Ye, Yuqing Lin, Siwen Wei

**Affiliations:** 1 Northwest Institute of Mechanical and Electrical Engineering, Xianyang, Shaanxi, China; 2 School of Aeronautics, Northwestern Polytechnical University, Xi’an, Shaanxi, China; 3 School of Information Engineering, Chang’an University, Xi’an, Shaanxi, China; 4 39th Research Institute of China Electronics Technology Group Corporation, Xi’an, Shaanxi, China; 5 Shaanxi Key Laboratory of Antenna and Control Technology, Xi’an, Shaanxi, China; Ariel University, UNITED KINGDOM OF GREAT BRITAIN AND NORTHERN IRELAND

## Abstract

Enhancing the resilience of Autonomous Unmanned Swarms (AUS) requires policies that remain effective under severe, structured disruptions while respecting the heterogeneous semantics of inter–subsystem interactions. Existing reinforcement learning (RL) approaches typically aggregate first–order neighborhoods in a path–agnostic manner, thereby blurring typed, ordered, and directed multi–hop dependencies encoded by domain meta–paths. We propose **MPGPD-RC**, a **M**eta- **P**ath **G**uided **P**olicy **D**istillation framework for **R**esilient **C**oordination that couples: (i) meta-path–guided embeddings learned by path-specific graph attention with contrastive reconstruction and attention fusion, and (ii) a teacher–student scheme in which a PPO teacher trained with a relaxed meta-path mask provides trajectories, and a student aligns both action distributions (KL) and trajectory-level structural codes via path-aware contrastive learning. Empirical evaluations validate that MPGPD-RC consistently surpasses state-of-the-art baselines across diverse perturbation scenarios by modeling complex, high-order dependencies that underpin resilient coordination.

## 1 Introduction

Autonomous Unmanned Swarm (AUS) [1,2] represents a transformative leap in multi-agent systems, integrating a diverse ensemble of autonomous platforms, including unmanned aerial vehicles (UAVs) [3], unmanned ground vehicles (UGVs) [4], and unmanned surface vessels (USVs) [5]. These sophisticated configurations exploit state-of-the-art real-time decision-making capabilities, advanced automation protocols, and heterogeneous network architectures that seamlessly interlink sensor arrays, computational intelligence modules, and robust communication infrastructures to execute complex collaborative tasks such as large-scale monitoring, dynamic environment mapping, and coordinated system behaviors [6–10]. This elevated level of autonomy and systemic inter-connectivity offers substantial advantages for high-demand, risk-sensitive applications, enhancing operational efficiency while reducing the need for constant human oversight.

Notwithstanding their profound capabilities, Autonomous Unmanned Swarms (AUS) exhibit intrinsic vulnerabilities stemming from their dependence on intricate and interdependent system architectures [11]. Disruptions such as signal interference, network breaches, and physical damage constitute significant risks by affecting pivotal nodes and communication links, thereby undermining overall system efficacy and continuity [12,13]. The highly integrated configuration of these systems amplifies the impact of localized failures, which can precipitate cascading degradations in global performance. Enhancing the resilience of AUS—specifically their ability to endure, adapt to, and recover from multifaceted disturbances—has become a critical imperative in both research and real-world deployment contexts [14].

Conventional resilience frameworks primarily rely on static redundancy strategies such as auxiliary components and predefined communication alternatives, which are insufficient for managing the dynamic complexity characteristic of modern large-scale distributed systems [15,16]. Such approaches frequently overlook the higher-order interdependencies and latent semantic associations embedded within heterogeneous networked systems, thereby constraining their capacity to deliver robust and enduring resilience across fluid and unpredictable operational scenarios.

**Fig 1:**
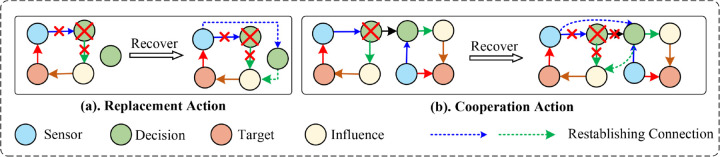
Depiction of two real time recovery strategies within a four class AUS configuration. **(a) Replacement action**: a compromised Decision node (marked with a red cross) is substituted with a standby node of equivalent classification. The original Sensor→Decision→Influencer→Target meta-path is reconstituted via reactivation of corresponding links (indicated by dashed blue and green arrows). **(b) Cooperation action**: in the absence of available redundant assets, operational continuity is achieved through dynamic rerouting across an alternative assemblage of functional nodes.

Fig 1 underscores the indispensable role of adaptive recovery mechanisms. Within a AUS network topology, four heterogeneous node categories are present: Sensor ($V_{S}$), Decision ($V_{D}$), Influencer ($V_{I}$), and Target ($V_{T}$). The failure of a single Decision node disrupts the Sensor–Decision–Influencer–Target–Sensor meta-path, which is vital for mission fulfillment. A meta-path denotes an ordered chain of distinct node types that encode semantic dependencies in the graph, thereby enabling multi-hop contextual reasoning. Traditional redundancy schemes rely on predefined standby nodes; restoring the disrupted path in such settings depends on quickly substituting the failed node with an equivalent component. If direct substitution proves impossible, task continuity must be maintained through rerouting operations across other available nodes. Both strategies demand timely evaluation and execution to minimize mission delays and avert cascading failures that static redundancy alone cannot absorb.

To address these limitations, a compelling direction for enhancing AUS resilience involves the deployment of reinforcement learning (RL) methodologies to facilitate adaptive recovery and systemic reconfiguration [17]. A salient advancement is presented by Sun et al. [14], who employed deep reinforcement learning (DRL) in conjunction with graph convolutional networks (GCNs) to dynamically optimize restorative actions. Through continuous policy refinement informed by environmental feedback, RL-driven agents possess the capability to autonomously reassign functional tasks or restructure coordination schemas upon anomaly detection, thereby contributing to the restoration of system integrity following disruptive incidents.

However, existing RL-based resilience methodologies are frequently constrained by practical deficiencies. Many approaches necessitate extensive exploration or retraining for each distinct failure scenario, rendering them unsuitable for deployment in real-time and safety-critical environments. A more fundamental shortcoming lies in the prevalent treatment of AUS as opaque entities during the learning process, thereby failing to incorporate domain-specific structural insights. The semantic richness of meta-paths, defined as the relational patterns and dependency trajectories among heterogeneous subsystems within a AUS, remains largely unexploited in prevailing learning frameworks [18]. The omission of these latent structural correlations often results in suboptimal recovery strategies and limited generalization across diverse disruption landscapes, as agents lack principled guidance regarding the coordination pathways most vital to sustaining resilience.

In addressing these challenges, this study proposes the MPGPD-RC framework. MPGPD-RC harnesses the meta-path semantics inherent in autonomous unmanned swarm (AUS) systems to inform a policy distillation mechanism that yields resilient coordination strategies. By embedding structural knowledge of inter-system interactions, MPGPD-RC steers the learning process toward preserving essential coordination links and functional pathways under conditions of component degradation or system-level disruptions. Meta-path representations function as inductive priors that enable reinforcement learning agents to emphasize and sustain critical relational configurations throughout both the training phase and online adaptation. The primary contributions of this research are as follows:

1. This study introduces a novel integration of meta-path-guided methodologies into representation learning to augment resilience in AUS, enabling the capture of complex multi-relational semantics within heterogeneous networks and facilitating the generation of semantically enriched and structurally expressive embeddings.

2. A meta-path-aware teacher-student reinforcement learning architecture is proposed to uphold action-level structural fidelity by preserving the teacher model’s topological coherence and AUS-specific semantic constructs through a retention-driven policy distillation process during inter-model knowledge transfer.

3. Empirical evaluations across a diverse set of disruption scenarios demonstrate that the proposed MPGPD-RC framework consistently attains superior performance benchmarks, surpassing existing state-of-the-art approaches in all examined contexts.

## 2 Related works

Recent progress in resilience augmentation for Autonomous Unmanned Swarms (AUS) has been primarily driven by three core methodological paradigms: mathematical optimization formulations, adaptive algorithmic strategies, and deep reinforcement learning (DRL)-based architectures. Although these approaches have yielded meaningful theoretical and empirical advancements, they exhibit intrinsic limitations in effectively capturing the dynamic and multifactorial complexities characteristic of highly variable and unpredictable system environments.

Mathematical optimization has constituted a foundational approach for formalizing and improving resilience within autonomous unmanned swarm (AUS) systems [19,20]. Such models typically define resilience through objective functions aimed at maximizing system reliability and minimizing recovery-related costs. Xu et al. [21] introduced a stochastic optimization framework designed to expedite the restoration of critical infrastructure networks, explicitly accounting for uncertainties in repair durations. Peiravi et al. advance a universal redundancy allocation model that systematically assigns spares and contingency elements to reinforce overall system reliability [22]. Similarly, Chen et al. [23] proposed a multi-stage optimization paradigm that incorporates evolutionary algorithms to derive Pareto-optimal strategies for enhancing the resilience of AUS in complex, dynamic environments.

Despite their theoretical rigor, optimization-based models frequently rely on highly accurate data inputs and entail substantial computational overhead, rendering them impractical for deployment in real-time operational contexts. Additionally, these models often utilize index-based heuristics, exemplified by the work of Zhang et al. [24], which prioritize system components based on predetermined indices. Such methodologies are prone to generating globally suboptimal outcomes due to their inability to account for the complex interdependencies inherent in heterogeneous network structures. These limitations constrain the applicability of optimization techniques in volatile and high-uncertainty environments, where agile and adaptive recovery capabilities are paramount [15]. Adaptability algorithms emphasize dynamic reconfiguration as a means of countering evolving disruptions and environmental variability in Autonomous Unmanned Swarm (AUS) systems. Qiang et al. [25] presented a resilience optimization framework tailored for multi-drone formations, facilitating real-time structural reconfiguration in response to system-level perturbations. Tran et al. [26] developed a stochastic node reconnection algorithm designed to reshape network architecture, thereby enhancing system robustness under disruptive conditions.

Although these algorithms contribute meaningful enhancements in terms of flexibility and resource efficiency, they predominantly depend on heuristic or rule-based strategies that exhibit inherent limitations in capturing the intricacies of complex and dynamic operational scenarios. The emergence of deep reinforcement learning (DRL) [27–30] has fundamentally transformed resilience methodologies by empowering autonomous agents to learn and make decisions within high-dimensional and complex operational landscapes. DRL-based architectures, particularly those integrating deep neural networks with reinforcement learning algorithms, have shown strong capabilities in exploring and optimizing the vast state-action spaces characteristic of autonomous unmanned swarms (AUS). Sun et al. [14] exemplified this advancement by fusing graph convolutional networks (GCNs) with proximal policy optimization (PPO) to enable the dynamic acquisition of recovery strategies, yielding marked enhancements in system resilience under disruptive conditions.

It is worth mentioning that Graph convolutional networks (GCNs) [31–34] have demonstrated strong efficacy in modeling structural dependencies within autonomous unmanned swarm (AUS) systems [14,17]. Peng et al. [35] illustrated the integration of GCNs within DRL frameworks to reinforce the resilience of IoT infrastructures against cyber disruptions. Dong et al. [36] employed GCN-augmented DRL for adaptive real-time resource allocation in large-scale distributed systems. Nevertheless, conventional DRL and GCN-based models predominantly concentrate on first-order node proximities, neglecting the higher-order relational dependencies that underpin the intricate, multi-hop dynamics inherent to AUS.

This gap motivates a shift from local adjacency–centric modeling to a semantics-aware framework grounded in meta-paths. Meta-paths [37], characterized as ordered sequences of node types connected via directed edges, serve as an effective construct for representing higher-order dependencies within heterogeneous networks [38,39]. By encapsulating multi-hop relational patterns, meta-paths enable a comprehensive depiction of systemic dynamics, thereby facilitating the fusion of local and global structural information [39]. This representational richness is particularly vital in the context of autonomous unmanned swarm (AUS) systems, where sensor, decision, communication, and actuator nodes engage in complex, hierarchical, and multi-phased interactions that demand an expressive and semantically-aware modeling framework.

Recent investigations have applied meta-path-based methodologies across diverse domains, yet their application to enhancing resilience in autonomous unmanned swarm (AUS) systems remains underexplored. Tran et al. [40] utilized meta-path constructs to examine information flow stability in networked infrastructures, with an emphasis on structural connectivity rather than adaptive resilience mechanisms. Sun et al. [14] incorporated first-order graph relations into DRL frameworks-CGPPO, which relies on GCN-based first-order neighborhood aggregation and stacked layers to expand receptive fields yet remains path-agnostic by isotropically mixing messages from heterogeneous relation types, directions, and hop orders, **MPGPD-RC** explicitly treats meta-paths as learnable relational templates with path-specific encoders and contrastive reconstruction to preserve typed, ordered, and directed multi-hop semantics, fuses the resulting meta-path representations via attention to emphasize salient coordination routes, constrains decision making through meta-path masks, and conducts path-aware contrastive distillation to align not only action distributions but also trajectory-level structural codes, thereby capturing role-consistent multi-hop dependencies and resilient backup routes that path-agnostic GCN–PPO baselines typically blur.

## 3 Markov decision process formulation

In this study, we formulate the resilience enhancement challenge for the AUS as a Markov Decision Process (MDP), represented by the tuple

$$\langle S,A,T,R,\gamma ,p(s_{0})\rangle ,$$ (1)

where the state space, action space, transition kernel, reward, discount factor, and initial distribution are defined below without relying on later sections.

Let G=(V,E) denote a directed heterogeneous graph, where *V* is the set of nodes and E⊆V×V represents the set of directed edges. Each node v∈V is assigned a categorical type as follows:

$$𝒯(v)\in \{V_{S},V_{D},V_{I},V_{T}\},$$ (2)

where $V_{S}$, $V_{D}$, $V_{I}$, and $V_{T}$ represent sensor, decision-making, influencer, and target nodes, respectively. These functional node types delineate the foundational operational roles embedded within the architectural framework of Autonomous Unmanned Swarm (AUS). AUS further define a set of meta-paths that formalize domain-specific interaction sequences among heterogeneous node types.

$$𝒫\;=\;\{\;P_{1},\;P_{2},\ldots ,\;P_{K}\},$$ (3)

where *L* represents the length of the meta-path and each $V_{i_{j}}$ is selected from $\left\{V_{S},V_{D},V_{I},V_{T}\right\}$. For any node *v*, the meta-path constrained neighbors are defined as:

$${𝒩}^{\left(k\right)}(v)=\left\{u\in V|(v,\ldots ,u)\text{ follows meta-path }P_{k}\right\}.$$ (4)

The meta-paths constitute a domain-informed library that abstracts operation-stage regularities commonly observed in AUS—namely sensing, decision-making, influence/execution, target engagement, and feedback—together with widely recurring patterns such as reconnaissance information sharing (sensor–sensor consensus), hierarchical decision coordination (multi-level decider interaction), and decision feedback (sensor–decider confirmation) [14,17,18,41]. Their explicit structures are:

$$P_{1}=V_{S}\;\to \;V_{D}\;\to \;V_{I}\;\to \;V_{T}\;\to \;V_{S},\;P_{2}=V_{S}\;\to \;V_{D}\;\to \;V_{D}\;\to \;V_{I}\;\to \;V_{T}\;\to \;V_{S},\;P_{3}=V_{S}\;\to \;V_{D}\;\to \;V_{S}\;\to \;V_{D}\;\to \;V_{I}\;\to \;V_{T}\;\to \;V_{S},\;P_{4}=V_{S}\;\to \;V_{S}\;\to \;V_{D}\;\to \;V_{I}\;\to \;V_{T}\;\to \;V_{S}.$$ (5)

For every node v∈V, we maintain a family of path-specific embeddings $\{h_{v}^{\left(k\right)}{\}}_{k=1}^{\left|P\right|}$ and their fused representation

$$h_{v}=\sum_{k=1}^{\left|P\right|}\beta_{k}\;h_{v}^{\left(k\right)},$$ (6)

where $\left\{\beta_{k}\right\}$ are learnable fusion weights.

**State space S.** At discrete time *t*, the global state $s_{t}\in S$ is an attention-weighted aggregation of the fused node embeddings:

$$s_{t}=\sum_{i=1}^{\left|V\right|}\alpha_{i}\;h_{v_{i}},$$ (7)

where $\left\{\alpha_{i}\right\}$ are attention coefficients derived from $\left\{h_{v_{i}}\right\}$. Thus *s*_*t*_ summarizes the heterogeneous, typed, and directed multi-hop information implied by P and {*E*_*k*_}.

**Action space A.** A decision specifies a *cooperation–replacement* pair $a_{t}=(c_{t},r_{t})$ drawn from $\left\{v_{1},\ldots ,v_{\left|G\right|}\}\cup \{Exit\right\}$, with the semantic constraint $c_{t}\neq r_{t}$ whenever both are valid nodes. The nominal action space is

$$A=\{(c,r)\;|\;c,r\in \{v_{1},\ldots ,v_{\left|G\right|}\}\cup \{Exit\}\}.$$ (8)

Feasibility is enforced by binary masks for cooperation and replacement, together with a hard meta-path mask

M(s,a)∈{0,1} (9)

that retains only actions consistent with at least one meta-path constraint derived from P and {*E*_*k*_}. For exploration we use a relaxed mask

$$\hat{M}(s,a)=M(s,a)+\lambda$$ (10)

with a small λ>0; probabilities are normalized within the policy.

**Transition kernel T.** Given *s*_*t*_ and $a_{t}=(c_{t},r_{t})$, the environment applies the selected recovery primitive to the current topology *G*_*t*_: (i) *replacement* substitutes a compromised node by the designated standby and reactivates incident typed links that realize at least one meta-path instance; (ii) *cooperation* reroutes along alternative functional nodes consistent with P. Denote the updated graph by

$$G_{t+1}=Apply(G_{t},a_{t}).$$ (11)

Path-specific neighborhoods and embeddings $\left\{h_{v}^{\left(k\right)}\right\}$ are recomputed on *G*_*t* + 1_, fused to $\left\{h_{v}\right\}$, and aggregated to yield *s*_*t* + 1_. Stochastic exogenous factors (e.g., new failures) are absorbed by

$$T(s_{t+1}\;|s_{t},a_{t}).$$ (12)

**Reward function *R*.** Let *r*_*t*_ denote the unshaped (base) environment reward at time step *t*, which reflects the immediate reward received by the system.

Following the resilience-assessment model proposed by prior studies [14,17,41,42], we define at each time step *t* an instantaneous resilience score *r*_*t*_ that mirrors their global metricR. Let *f*_*t*_ denote the current operational performance of the AUS; *f*_*b*_ the mission-baseline performance; *f*_0_ the pre-attack performance at *t*_0_; *f*_*m*_ the minimum performance reached at *t*_*m*_ after the attack at *t*_*a*_; *t*_*r*_ the restoration time when the performance first returns to (or surpasses) *f*_*b*_; and *t*_*e*_ the end of the evaluation horizon.

The mean post-recovery performance over the interval $\left[t_{r},t_{e}\right]$ is

$$f_{r}=(t_{e}-t_{r}+1{)}^{-1}\sum_{k=t_{r}}^{t_{e}}f_{k}.$$ (13)

The normalised recovery-time factor is

$$\eta =\frac{t_{r}-t_{0}}{t_{e}-t_{0}},$$ (14)

and the completion factor is

$$\zeta =C_{m}(f_{r}/f_{b})\;1_{f_{r}\geq f_{b}}$$ (15)

with importance weight *C*_*m*_>0 and indicator 1. Using these quantities, the instantaneous score is

$$r_{t}=\frac{\min(f_{t},f_{b})}{f_{b}}\;\frac{f_{r}}{f_{b}}\;[\frac{f_{m}}{f_{0}}+1-\eta \;\left(\frac{f_{r}}{f_{b}}-\frac{f_{m}}{f_{b}}\right)+\zeta ],$$ (16)

where larger values signify a state that is closer to the desired resilient behaviour—high performance, rapid recovery, and stable operation. This scalar is inserted into the shaped reward:

$$r_{t}^{\prime }=\left\{ \begin{array}{cc} r_{t}+\delta 𝒞(v_{i},v_{j}), & \text{if a replacement/cooperative occurs},\\ 0, & \text{otherwise}. \end{array}\right.$$ (17)

To accommodate the semantic heterogeneity inherent in evaluating collaborative efficiency across diverse meta-paths in AUS, we introduce a meta-path-aware similarity metric

$$𝒞(v_{i},v_{j})=\sum_{k=1}^{\left|𝒫\right|}\beta_{k}\cdot \text{sim}\left(h_{v_{i}}^{\left(k\right)},h_{v_{j}}^{\left(k\right)}\right),$$ (18)

where $h_{v_{i}}^{\left(k\right)}$ and $h_{v_{j}}^{\left(k\right)}$ denote the embeddings of nodes $v_{i}$ and $v_{j}$ specific to meta-path *P*_*k*_, as defined in Eq (6). The similarity function is cosine similarity:

$$\text{sim}(u,v)=\frac{u^{⊤}v}{\left\|u\|\|v\right\|}.$$ (19)

The weights $\beta_{k}$ are shared with those used in Eq (6), ensuring consistent meta-path importance across embedding fusion and collaborative assessment.

**Discounting and return.** Let γ∈(0,1) be the discount factor. The *discounted return* from time *t* is

$$G_{t}\;=\;\sum_{k=0}^{\infty }\gamma^{k}\;R(s_{t+k},a_{t+k},s_{t+k+1}),$$ (20)

which balances immediate recovery benefits with long-horizon resilient performance.

**Policies and objective.** A policy π(·∣s) is a stochastic kernel on A given s∈S. Over the initial-state distribution *p*(*s*_0_), the control objective is

$$\pi^{⋆}\;\in \;\arg\max_{\pi }\;{𝔼}_{\;s_{0}\sim p,\;a_{t}\sim \pi (\cdot |s_{t}),\;s_{t+1}\sim T(\cdot |s_{t},a_{t})}[\;G_{0}\;].$$ (21)

This optimal policy satisfies the Bellman optimality condition:

$$V^{⋆}(s)=\max_{a\in A}\;{𝔼}_{s^{\prime }\sim T(\cdot |s,a)}[\;R(s,a,s^{\prime })+\gamma V^{⋆}(s^{\prime })\;],$$ (22)

$$Q^{⋆}(s,a)={𝔼}_{s^{\prime }\sim T(\cdot |s,a)}[\;R(s,a,s^{\prime })+\gamma \max_{a^{\prime }}Q^{⋆}(s^{\prime },a^{\prime })\;].$$ (23)

## 4 Method

### 4.1 Ethics statement

No ethical approval was required for this study because the research involved only publicly available anonymized data and did not involve human participants, identifiable human material, or animals.

**Fig 2:**
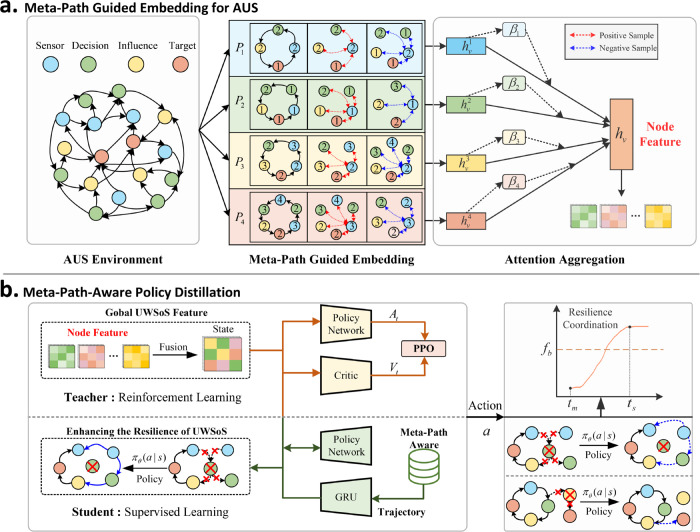
The proposed Meta- Path Guided Policy Distillation for Resilient Coordination (MPGPD-RC) framework comprises two interdependent modules: **(a) Meta-Path Guided Embedding**, responsible for encoding heterogeneous inter-node relationships into semantically rich and structurally aware representations, and **(b) Meta-Path-Aware Policy Distillation**, which facilitates the transfer of expert-level decision policies from a teacher model to a computationally efficient student model while maintaining the structural semantics intrinsic to the AUS topology.

The MPGPD-RC framework, as depicted in Fig 2, integrates two interdependent modules: (a) Meta-Path Guided Embedding and (b) Meta-Path-Aware Policy Distillation. The process begins with the generation of multi-granular node embeddings using meta-path-constrained graph attention networks (GATs), where each embedding encodes interaction schemas reflective of the heterogeneous AUS topology. These path-specific embeddings are subsequently fused through an attention-based mechanism to form a comprehensive global state vector, which encapsulates the structural semantics of the AUS network. This global state vector is then used as a common input for both the teacher and student policy networks. The teacher policy is optimized through Proximal Policy Optimization (PPO) under relaxed meta-path constraints, generating high-fidelity trajectories that align with the AUS’s structural intricacies. Concurrently, the student policy is trained via supervised learning, employing a dual-channel distillation approach that minimizes Kullback-Leibler (KL) divergence between action distributions and enforces structural coherence by contrastively aligning trajectory embeddings encoded by a GRU.

### 4.2 Meta-path guided embedding for AUS

Meta-Path Guided Embedding depicts the embedding moudule comprising three sequential stages: (1) meta-path–constrained traversal identifies semantically salient node sequences reflective of heterogeneous relational contexts, (2) path-specific graph attention networks (GATs), enhanced by contrastive sampling strategies, learn distinct embeddings for each meta-path to capture contextual dependencies, (3) an attention-based aggregation mechanism integrates the resulting embeddings into a unified node representation that encapsulates both localized structural attributes and higher-order semantic correlations.

For each meta-path *P*_*k*_, a meta-path-specific embedding $h_{v}^{\left(k\right)}\in {\mathbb{R}}^{d}$ is computed for node *v*. Let $h_{v}^{\left(k,l\right)}$ denote the embedding of node *v* at layer *l*, with 1≤l≤L. At each layer *l*, node *v* aggregates messages from its meta-path-specific neighborhood ${𝒩}^{\left(k\right)}(v)$. Denote $h_{u}^{\left(k,l-1\right)}$ as the embedding of neighbor node *u* from the previous layer (*l*–1). The message propagated from *u* to *v* is defined as:

$$m_{v\leftarrow u}^{\left(k,l\right)}=\phi \left(W_{k}^{\left(l\right)}h_{u}^{\left(k,l-1\right)}+b_{k}^{\left(l\right)}\right),$$ (24)

where $W_{k}^{\left(l\right)}\in {\mathbb{R}}^{d\times d}$ and $b_{k}^{\left(l\right)}\in {\mathbb{R}}^{d}$ are trainable parameters associated with meta-path *P*_*k*_ at layer *l*, and ϕ(·) denotes a ReLU activation function. After computing the set of incoming messages $\left\{m_{v\leftarrow u}^{\left(k,l\right)}\right\}$, node *v* aggregates these messages to update its embedding as follows:

$$e_{vu}^{\left(k,l\right)}=\text{LeakyReLU}\left(𝐚k^{(l)⊤}\left[W{Q,k}^{\left(l\right)}h_{v}^{\left(k,l-1\right)}\|W_{K,k}^{\left(l\right)}h_{u}^{\left(k,l-1\right)}\right]\right),$$ (25)

$$\alpha_{vu}^{\left(k,l\right)}=\frac{\exp\left(e_{vu}^{\left(k,l\right)}\right)}{\sum_{u^{\prime }\in {𝒩}^{\left(k\right)}(v)}\exp\left(e_{vu^{\prime }}^{\left(k,l\right)}\right)}.$$ (26)

$$h_{v}^{\left(k\right)}=h_{v}^{\left(k,l\right)}=\psi \left(\sum_{u\in {𝒩}^{\left(k\right)}(v)}\alpha_{vu}^{\left(k,l\right)}m_{v\leftarrow u}^{\left(k,l\right)}\right),$$ (27)

where ψ(·) represents the ELU activation function applied for nonlinear transformation, $\alpha_{vu}^{\left(k,l\right)}\in [0,1]$ denotes the attention coefficient capturing the relative importance of neighbor *u* to node *v* under meta-path *P*_*k*_ at layer *l*, $W_{Q,k}^{\left(l\right)},W_{K,k}^{\left(l\right)}\in {\mathbb{R}}^{d\times d}$ are the meta-path-specific query and key projection matrices utilized in Eq (25), and $a_{k}^{\left(l\right)}\in {\mathbb{R}}^{2d}$ is the learnable attention vector corresponding to meta-path *P*_*k*_ at layer *l*.

After deriving the meta-path-specific embeddings $h_{v}^{\left(k\right)}$ for each $P_{k}\in 𝒫$, a unified node representation is obtained by computing a weighted summation over the set $\{h_{v}^{\left(k\right)}{\}}_{k=1}^{\left|𝒫\right|}$. Formally,

$$h_{v}=\sum_{k=1}^{\left|𝒫\right|}\beta_{k}\;h_{v}^{\left(k\right)},$$ (28)

where each scalar $\beta_{k}$ signifies the relative contribution of the *k*-th meta-path to the composite embedding of node *v*.

$$\beta_{k}=\frac{\exp\left(q^{⊤}\tanh\left(W_{k}^{\left(f\right)}h_{v}^{\left(k\right)}+b_{k}^{\left(f\right)}\right)\right)}{\sum_{m=1}^{\left|𝒫\right|}\exp\left(q^{⊤}\tanh\left(W_{m}^{\left(f\right)}h_{v}^{\left(m\right)}+b_{m}^{\left(f\right)}\right)\right)},$$ (29)

where $W_{k}^{\left(f\right)}\in {\mathbb{R}}^{d\times d}$, $b_{k}^{\left(f\right)}\in {\mathbb{R}}^{d}$, and $q\in {\mathbb{R}}^{d}$ are trainable parameters.

To ensure that $h_{v}^{\left(k\right)}$ encapsulates the structural dependencies governed by meta-path *P*_*k*_, we define an induced edge set $E_{k}\subseteq V$ × V. Specifically, *E*_*k*_ is constructed by enumerating all valid path instances conforming to the schema of *P*_*k*_. Formally, we define:

$$E_{k}=\left\{(v,u)\;|\;\exists \;(v=v_{0}\overset{r_{1}}{\to }v_{1}\overset{r_{2}}{\to }\cdots \overset{r_{m}}{\to }v_{m}=u),\;(r_{1}r_{2}\cdots r_{m})\in P_{k}\right\},$$ (30)

where each edge $(v,u)\in E_{k}$ indicates that there exists at least one path instance from node *v* to node *u* whose relation-type sequence matches the meta-path *P*_*k*_. Thus, *E*_*k*_ captures the set of node pairs semantically connected under *P*_*k*_.

The objective is to enforce high similarity $h_{v}^{(k)⊤}h_{u}^{\left(k\right)}$ approaching  + 1 for $(v,u)\in E_{k}$, and low similarity approaching –1 for $(v,u)\notin E_{k}$. Formally, we define:

$${ℒ}_{k}=-\sum_{(v,u)\in E_{k}}\log\sigma \left(h_{v}^{(k)⊤}h_{u}^{\left(k\right)}\right)-\sum_{(v,u)\notin E_{k}}\log\left(1-\sigma \left(h_{v}^{(k)⊤}h_{u}^{\left(k\right)}\right)\right),$$ (31)

where σ(·) is the sigmoid function, which bounds the inner product between (–1) and  + 1. The first summation rewards cases where *v* and *u* are deemed connected by *P*_*k*_ by encouraging $\sigma (h_{v}^{(k)⊤}h_{u}^{\left(k\right)})\approx 1$. The second summation penalizes pairs $(v,u)\notin E_{k}$, encouraging $\sigma (h_{v}^{(k)⊤}h_{u}^{\left(k\right)})\approx 0$. Aggregating ${ℒ}_{k}$ across all meta-paths k=1,…,|𝒫| yields the complete unsupervised embedding loss:

$${ℒ}_{embed}=\sum_{k=1}^{\left|𝒫\right|}{ℒ}_{k}.$$ (32)

Minimization of ${ℒ}_{embed}$ guarantees that each meta-path-specific embedding $h_{v}^{\left(k\right)}$ reconstructs the relational dependencies defined by its corresponding meta-path *P*_*k*_.

By restricting message passing to domain-specific meta-paths, learning path-specific attention encoders under contrastive regularization, and fusing the resulting representations through data-driven saliency weights, the embedding module disentangles typed, ordered, and direction-aware interaction channels that extend beyond first-hop adjacency, suppresses spurious correlations arising from heterogeneous neighborhoods, and adaptively emphasizes the coordination routes most predictive of resilient behavior.

### 4.3 Meta-path-aware policy distillation

Fig 2(b) illustrates the distillation pipeline. Given the global state *s*_*t*_, synthesized from the fused node embeddings, the teacher policy in the upper branch is optimized using Proximal Policy Optimization (PPO) under relaxed meta-path constraints, producing advantage signals *A*_*t*_ and value estimations $V_{t}$. These teacher-generated trajectories are processed by the student branch, wherein a GRU-based encoder models the sequential dependencies of meta-path transitions. The student policy $\pi_{\theta }$ is trained via a dual-objective scheme incorporating path-sensitive contrastive loss and Kullback–Leibler (KL) divergence, thereby enabling the student to emulate the teacher’s behavior while preserving the structural semantics imposed by the meta-paths.

**Teacher Policy with PPO:** The teacher policy $\pi_{\Theta }(a|s_{t})$ interfaces with the environment to generate high-quality decision trajectories, which serve as optimal or near-optimal behavioral references for student policy distillation. Training of the teacher is performed using Proximal Policy Optimization (PPO). At each decision point *s*_*t*_, the teacher outputs a nominal policy distribution $\pi_{\Theta }(a|s_{t})$ over the admissible action space $𝒜(s_{t})$. The resulting relaxed policy $\pi_{\Theta }^{\left(\text{relaxed}\right)}(a|s_{t})$ is defined as:

$$\pi_{\Theta }^{\left(\text{relaxed}\right)}(a|s_{t})=\frac{\pi_{\Theta }(a|s_{t})\;\hat{ℳ}(s_{t},a)}{\sum_{a^{\prime }\in 𝒜(s_{t})}\pi_{\Theta }(a^{\prime }|s_{t})\;\hat{ℳ}(s_{t},a^{\prime })}.$$ (33)

The teacher is trained using PPO with the objective:

$$\ell_{t}(\Theta )=-\min\left[r_{t}(\Theta )A_{t},\mathrm{clip}\left(r_{t}(\Theta ),1-\epsilon ,1+\epsilon \right)A_{t}\right].$$ (34)

where $r_{t}(\Theta )$ is the probability ratio defined by:

$$r_{t}(\Theta )=\frac{\pi_{\Theta }^{\prime }(a_{t}|s_{t})}{\pi_{\Theta_{old}}^{\prime }(a_{t}|s_{t})},$$ (35)

and *A*_*t*_ is the advantage function. This ensures stable training of the teacher, while ensuring a balance between exploration and exploitation.

Every *K* training epochs, new trajectories are generated using the updated teacher policy $\pi_{\Theta }$, and 25% of the outdated samples in the experience buffer 𝒟 are replaced to ensure data freshness and policy relevance.

**Student policy:** The student policy $\pi_{\theta }(a|s_{t})$ is trained to replicate the behavioral patterns of the teacher policy through two principal learning objectives:

1. Knowledge Distillation (KD): The student seeks to approximate the teacher’s softened action distribution, learning from the probabilistic outputs of the teacher policy to mimic its decision-making strategy.

2. Path-Aware Contrastive Learning (PaC): The student is trained to preserve the structural semantics captured in the teacher’s trajectories, with particular emphasis on aligning meta-path-induced relational patterns.

To encode the temporal and structural dynamics of the teacher’s trajectories, a GRU (Gated Recurrent Unit) encoder is employed to process the sequence of state-action pairs. This sequence is transformed into a fixed-dimensional latent representation $c_{\tau }$, referred to as the trajectory code, which serves as a compact descriptor for contrastive alignment and policy distillation.

Let *z*_*t*_ denote the concatenated vector representing the meta-path-specific encoding of the trajectory at time step *t*. The meta-path embedding for step *t* is formally defined as:

$$z_{t}=[h_{v_{t}}^{\left(1\right)}\|\ldots \|h_{v_{t}}^{\left(|𝒫|\right)}]⊙e_{P(\tau ,t)}\in {\mathbb{R}}^{Kd},$$ (36)

where $h_{v_{t}}^{\left(k\right)}$ denotes the embedding of node $v_{t}$ along meta-path *P*_*k*_. The vector $e_{P(\tau ,t)}\in \{0,1{\}}^{\left|𝒫\right|}$ is a one-hot indicator that activates the meta-path applied at time *t*. More precisely, its *k*-th component is defined as:

$$e_{P(\tau ,t)}^{\left(k\right)}=1\;\left[\;P(\tau ,t)=P_{k}\;\right],$$ (37)

where P(τ,t) denotes the meta-path governing the decision at step *t* of trajectory τ, and 1[·] is the indicator function. The element-wise product ⊙ ensures that only the segment of the concatenated representation corresponding to the active meta-path contributes to the step-wise trajectory descriptor, while all other segments are masked out.

The GRU integrates the current input *z*_*t*_ and the previous hidden state ${𝐡}_{t-1}$ to compute the new hidden state ${𝐡}_{t}$. It employs update and reset gates to control the flow of information, and the final hidden state is a combination of the previous state and the updated information. The equations for this process are:

$${𝐡}_{t}=(1-{𝐮}_{t})⊙{𝐡}_{t-1}+{𝐮}_{t}⊙\tanh\left(W_{h}z_{t}+U_{h}({𝐫}_{t}⊙{𝐡}_{t-1})+{𝐛}_{h}\right),$$ (38)

where ${𝐮}_{t}$ and ${𝐫}_{t}$ are the update and reset gates, respectively, and σ(·) and tanh(·) are the sigmoid and hyperbolic tangent activations.

The final output of the GRU at time step *T* is defined as the trajectory code:

$$c_{\tau }={𝐡}_{T}\in {\mathbb{R}}^{d_{c}},$$ (39)

where *d*_*c*_ denotes the dimensionality of the GRU hidden state. This trajectory code $c_{\tau }$ encapsulates the temporal evolution and structural semantics of the teacher’s decision sequence, serving as a condensed representation to supervise and inform the student policy’s learning process.

The student policy is trained not only to replicate the teacher’s action distribution but also to internalize the structural regularities captured in the teacher’s trajectory embeddings. The path-aware contrastive loss enforces structural alignment by minimizing the divergence between the teacher’s and student’s trajectory encodings:

$${ℒ}_{PaC}=-\frac{1}{B}\sum_{i=1}^{B}\log\frac{\exp\left(\mathrm{sim}(c_{i}^{+},c_{i}^{-})/\tau_{c}\right)}{\sum_{j=1}^{B}\exp\left(\mathrm{sim}(c_{i}^{+},c_{j}^{-})/\tau_{c}\right)},$$ (40)

where $\mathrm{sim}(u,v)=\frac{u^{⊤}v}{\left\|u\|\|v\right\|}$ denotes cosine similarity, $c_{i}^{+}$ and $c_{i}^{-}$ represent the teacher and student trajectory encodings of the same trajectory $\tau_{i}$, and $\tau_{c}$ is a temperature hyperparameter that modulates the sensitivity of the similarity distribution.

In Eq 40, the pair $\left(c_{i}^{+},c_{i}^{-}\right)$ constitutes the positive sample, while all other student encodings $c_{j}^{-}$ with j≠i within the same batch serve as negatives. This design ensures that each teacher trajectory embedding is pulled closer to its student counterpart while being contrasted against embeddings from other trajectories, thereby enhancing both alignment and discriminability.

In parallel, the knowledge distillation loss compels the student policy to approximate the teacher’s action distribution. This alignment is enforced through a Kullback–Leibler divergence objective:

$${ℒ}_{KD}={𝔼}_{s\sim 𝒟}\left[D_{KL}\left(\pi_{\Theta }^{\left(s\right)}(\cdot |s)\|\pi_{\theta }^{\left(s\right)}(\cdot |s)\right)\right].$$ (41)

This formulation guides the student toward replicating the teacher’s decision behavior while preserving computational tractability and training stability.

The student policy $\pi_{\theta }(a|s_{t})$ is optimized through a structured training pipeline consisting of sequential stages. First, a batch of trajectories $\{\tau_{i}{\}}_{i=1}^{B}$ is uniformly sampled from the teacher’s experience buffer 𝒟, providing exposure to a diverse set of meta-path-constrained decision sequences. The learning objective integrates both decision-level imitation and structural consistency through the following composite loss function:

$${ℒ}_{\text{total}}=\alpha {ℒ}_{\text{KD}}+\beta {ℒ}_{\text{PaC}},$$ (42)

where α and β are weighting coefficients balancing the contributions of knowledge distillation and path-aware contrastive learning respectively.

The proposed meta-path guided teacher-student policy learning framework, delineated in Algorithm 1, facilitates a synergistic interaction between structured exploration driven by the teacher and efficient policy imitation performed by the student. The teacher policy $\pi_{\Theta }$, trained using Proximal Policy Optimization (PPO) under meta-path constraints (Eq (33)), yields high-quality trajectories that embed both task-specific reward signals and meta-path-informed collaborative semantics. These trajectories are incrementally transferred to the student policy $\pi_{\theta }$ through the dual-objective loss ${ℒ}_{\text{total}}$, which jointly enforces action distribution fidelity and structural alignment with meta-path relational patterns.

**Algorithm 1 Meta-path guided teacher-student policy learning.**

**Require:** Heterogeneous AUS graph G=(V,E), meta-path set 𝒫, temperature $\tau_{c}$, distillation weight λ

**Ensure:** Student policy $\pi_{\theta }$

1: **Initialize:** Teacher policy $\pi_{\Theta }$ with PPO, student policy $\pi_{\theta }$ with GRU encoder, experience buffer 𝒟←∅

2: **while** not converged **do**

3:   *// Teacher Policy Rollout*

4:   **for** each episode **do**

5:    Generate trajectory τ using Eq (33) with meta-path mask $\hat{ℳ}$

6:    Compute shaped reward $r_{t}^{\prime }$

7:   **end for**

8:   Update teacher Θ via PPO objective (Eq (34))

9:   *// Student Policy Distillation*

10:   **for** $N_{\text{student}}$ steps **do**

11:    Sample batch $\{\tau_{i}\}\sim 𝒟$

12:    Encode trajectories $\left\{c_{\tau }\right\}$ via GRU

13:    Compute ${ℒ}_{\text{total}}=\alpha {ℒ}_{\text{KD}}+\beta {ℒ}_{\text{PaC}}$

14:    Update $\theta \leftarrow \theta -\eta \nabla_{\theta }{ℒ}_{\text{total}}$ with cosine-annealed η

15:   **end for**

16:   **if** tmodK=0 **then**

17:    Replace 25% of 𝒟 with new $\pi_{\Theta }$-generated trajectories

18:   **end if**

19: **end while**

## 5 Experiments

### 5.1 Experimental setup

**Fig 3:**
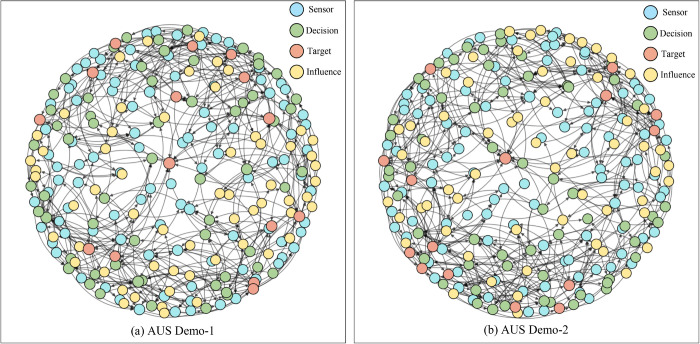
Illustration of the AUS network topology for demo1 and demo2. The system includes four types of entities: Sensor, Decision, Communication, and Actuator.

Following the settings in Refs [14,17,18,41,43–45], we evaluate the efficacy and resilience of the proposed RL-driven Meta-Path Optimization (MPGPD-RC) framework through extensive experiments conducted in a simulated autonomous unmanned swarm (AUS) environment. As illustrated in Fig 3, the AUS topology comprises four heterogeneous node types: Sensor, Decision, Influence, and Target. These nodes are interconnected via directed edges that encode the functional dependencies critical to the system’s operational workflow.

In accordance with the methodologies outlined in Refs [14,17,18,41,43–45], we employed NetworkX version 3.2.1 [46] for the construction of the AUS graph and utilized PyTorch version 2.2.2 for model training. As depicted in Fig 3, the AUS topology consists of four distinct node types: sensors ($V_{S}$), decision-making units ($V_{D}$), influencers ($V_{I}$), and targets ($V_{T}$). These nodes are interconnected through directed edges that represent the functional dependencies critical to the operational workflow of the system. Table 1 presents a summary of the key structural attributes of the AUS Demo network, which serves as the experimental basis for evaluating the MPGPD-RC framework. The network comprises a total of 185 nodes, interconnected by 399 directed edges, each encoding essential functional dependencies and communication pathways necessary to maintain the integrity and coherence of the system.

**Table 1:** Basic properties of AUS demo.

Node Type		Edge Type			
Type	Number	Type	Number	Type	Number
**V**	**185**	**E**	**399**	** $V_{T}\to V_{S}$ **	**53**
** $V_{S}$ **	**71**	** $V_{S}\to V_{D}$ **	**73**	** $V_{D}\to V_{S}$ **	**56**
** $V_{D}$ **	**45**	** $V_{S}\to V_{S}$ **	**75**	**-**	**-**
** $V_{I}$ **	**60**	** $V_{D}\to V_{I}$ **	**72**	**-**	**-**
** $V_{T}$ **	**10**	** $V_{I}\to V_{T}$ **	**70**	**-**	**-**

The experimental evaluation of the MPGPD-RC embedding was carried out under configured settings, covering both embedding generation and training procedures. Meta-path-guided embeddings were computed using a hierarchical graph attention network with a fixed embedding dimensionality of 128. Contrastive sampling was regulated by a temperature parameter of 0.1 to effectively differentiate between positive and negative node pairs. The embedding module was trained using the AdamW optimizer, initialized with a learning rate of 0.001, a batch size of 64, and executed over 500 training epochs.

The teacher policy was trained using Proximal Policy Optimization (PPO) with relaxed meta-path masks, employing an actor–critic architecture characterized by a learning rate of 1 × 10^−4^, a discount factor γ=0.97, and a generalized advantage estimation (GAE) parameter λ=0.9. Teacher rollouts produced advantage estimates *A*_*t*_ and value targets $V_{t}$, serving as supervisory signals for the student policy. The student policy incorporated a single-layer GRU encoder with a hidden state dimension of 128 to model the sequential dependencies of meta-path transitions. The training objective combined Kullback–Leibler divergence and path-aware contrastive learning, with respective weighting coefficients α=0.5 and β=0.25, these values are kept constant across all experiments to ensure fair comparability. Optimization was performed using the AdamW algorithm with an initial learning rate of 1 × 10^−4^, a batch size of 64, and the training process spanned 500 epochs.

To evaluate the robustness of the MPD-RL framework, we simulate four distinct adversarial scenarios, each targeting specific node combinations within the AUS environment. Following prior studies [14,17,18,41], we model each attack as an instantaneous removal of the targeted node(s), rather than gradual functionality degradation. The attacked nodes are selected uniformly at random within the first ten timestamps, ensuring that disruptions occur early in the mission and force the framework to demonstrate recovery under stringent conditions. The training process was conducted on NVIDIA A100 Tensor Core GPUs.

**SA (Sensor Attack):** This case simulates the adversarial compromise of Sensor ($V_{S}$) nodes, which are tasked with acquiring environmental information essential to system perception.

**DA (Decision Attack):** In this case, the attack is directed at Decision ($V_{D}$) nodes, the core computational entities that transform sensory data into executable commands. Failure at this stage critically disrupts the decision-making chain, offering a rigorous evaluation of the system’s robustness against cognitive impairment.

**IA (Influencer Attack):** Here, the focus of the attack shifts to Influencer ($V_{I}$) nodes, which directly govern behavioral adaptation and strategic responses of the Target nodes.

**MA (Multi-Node Attack):** This case represents a coordinated adversarial operation against all four categories—Sensor, Decision, Influencer, and Target—posing the most severe stress test by inducing global systemic disruption.

### 5.2 Experimental analysis

In this subsection, we present a detailed performance evaluation of the MPGPD-RC framework under the previously defined node disruption scenarios. The analysis emphasizes the individual and collective contributions of the framework’s components to the overall resilience ℛ of the AUS. To contextualize the effectiveness of MPGPD-RC, comparative benchmarking was conducted against several state-of-the-art baseline methods [14,17]. The baseline algorithms are summarized as follows:

- **Self-Resistance (SR):** Assesses the intrinsic resilience of the AUS without the incorporation of post-attack recovery mechanisms.
- **Random Node Reconnection (RN) [14]:** Implements random reconnections of disrupted nodes to functional ones, subject to capacity and edge-type constraints.
- **Maximum Degree Node Reconnection (MDN):** Prioritizes reconnection based on node degree centrality, favoring nodes with higher connectivity to improve system robustness.
- **CGPPO Algorithm (CGPPO) [14]:** Integrates Graph Convolutional Networks (GCNs) with Proximal Policy Optimization (PPO) within a reinforcement learning framework to optimize recovery strategies.
- **Genetic Algorithm-Based Multi-Swarm Cooperative Reconfiguration (GA) [43]:** Utilizes a Genetic Algorithm to iteratively optimize cooperative reconfiguration strategies for enhancing AUS resilience.
- **Kill Chain Optimization Method (KCOM) [11]:** Focuses on optimizing the observation, positioning, decision-making, and execution phases within the kill chain for improved system resilience.
- **Enhanced-Resilience Multi-Layer Attention GCN (EGCN) [18]:** Combines multi-layer attention mechanisms and regularization techniques to effectively model both global and local graph structures, enhancing resilience.

Table 2 presents a comparative analysis of the resilience metric ℛ achieved by MPGPD-RC and seven baseline algorithms across 12 disruption scenarios. The proposed framework consistently delivers the highest resilience scores across all disruption levels (25%, 50%, 75%), with performance differentials becoming more pronounced as attack intensity escalates. While methods such as EGCN and CGPPO incorporate graph neural network modules, they lack the capacity to extract and exploit deep structural semantics to the extent enabled by MPGPD-RC. This limitation undermines their ability to formulate resilient coordination policies under system-level stress. In the MA-50 % scenario, MPGPD-RC attains a resilience of 0.813, outperforming EGCN’s 0.779 by 4.2 %, a gain rooted in its capacity to explicitly encode and exploit inter-node collaboration chains. Under DA-50 % conditions, our framework achieves a resilience of 0.684—5.4 % above KCOM’s 0.637—by dynamically distilling and preserving the system’s functional hierarchies. MPGPD-RC, by incorporating meta-path-based semantics into its policy learning and decision-making processes, sustains consistently higher resilience, exhibiting performance gains of approximately 3% to 6% over EGCN and CGPPO under IA scenarios.

**Table 2:** Performance comparison in terms of resilience ℛ across four attack scenarios with different nodes attacked. The highest resilience in each scenario is highlighted in bold.

Method	SR	RN	MDN	GA	CGPPO	KCOM	EGCN	Our
**SA(25%)**	0.775	0.797	0.843	0.851	0.887	0.879	0.890	**0.907**
**DA(25%)**	0.723	0.744	0.763	0.782	0.811	0.799	0.807	**0.826**
**IA(25%)**	0.717	0.733	0.745	0.751	0.771	0.770	0.769	**0.789**
**MA(25%)**	0.789	0.810	0.833	0.901	0.919	0.916	0.920	**0.933**
**SA(50%)**	0.559	0.583	0.647	0.730	0.777	0.732	0.779	**0.798**
**DA(50%)**	0.487	0.507	0.544	0.631	0.661	0.637	0.659	**0.684**
**IA(50%)**	0.398	0.431	0.467	0.577	0.611	0.590	0.609	**0.635**
**MA(50%)**	0.561	0.583	0.633	0.721	0.779	0.761	0.780	**0.813**
**SA(75%)**	0.277	0.293	0.301	0.342	0.377	0.369	0.372	**0.409**
**DA(75%)**	0.259	0.267	0.260	0.363	0.390	0.379	0.388	**0.413**
**IA(75%)**	0.165	0.167	0.169	0.293	0.347	0.336	0.341	**0.377**
**MA(75%)**	0.299	0.321	0.349	0.393	0.447	0.438	0.452	**0.497**

### 5.3 Robust study

**Fig 4:**
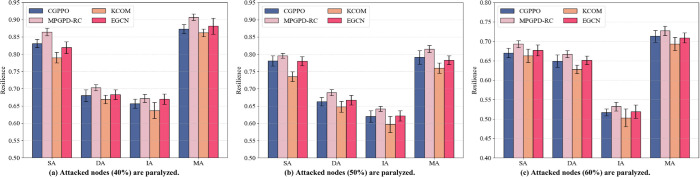
Robustness to random initial conditions. For each damage ratio (40%–60%) we re-initialise the AUS topology fifty times and report the mean resilience (bars) with one-standard-deviation whiskers across the four attack modes.

We next examine the sensitivity of each baseline to stochastic variations in the initial AUS state—a critical consideration for real-world deployments. For each damage ratio ranging from 40% to 60%, we generate fifty distinct network realizations, retrain every method to convergence, and report the mean final resilience along with its associated standard deviation (±1σ). Fig 4 presents the aggregated outcomes in the form of grouped bar charts: error whiskers capture the across-run variability in performance.

In trials with repeated initializations, the disparity in performance between the two leading baselines—EGCN and CGPPO—becomes increasingly evident. EGCN employs a deep architecture of traditional GCN layers followed by a single actor-critic head. When the graph spectrum is disrupted through stochastic rewiring, the multi-hop propagation exacerbates feature over-smoothing, resulting in a highly unstable gradient landscape. Although the overall performance may appear competitive under certain conditions (e.g., IA with 40% damage), the variance across runs is notably higher.

On the other hand, CGPPO partially mitigates this instability by incorporating CARCI-guided trajectories. However, it still depends on online learning of node representations and optimizes based on a fixed scalar reward. As a result, its convergence target tends to shift in response to changes in the initial topology—especially when particular structural configurations disproportionately favor certain resilience aspects (e.g., rapid recovery under SA vs. steady-state stability in MA). This leads to a consistent performance gap when compared to the proposed framework.

The MPGPD-RC method, which incorporates both Meta-Path Aware Policy Distillation and robust reinforcement learning strategies, outperforms the other baselines in terms of both convergence stability and long-term performance consistency. As evidenced by the results in Fig 4, our approach consistently achieves the highest resilience values across different attack scenarios, especially under conditions of higher damage (50% and 60%). The proposed method’s robustness to initial state variability arises from its ability to leverage meta-path-guided structural learning, which provides a more comprehensive understanding of the global topology and long-range dependencies. These results demonstrate the advantage of incorporating global graph semantics into policy distillation, enabling the proposed method to consistently deliver superior performance and greater resilience under dynamic and unpredictable real-world conditions.

### 5.4 Hyperparameters analysis

**Fig 5:**
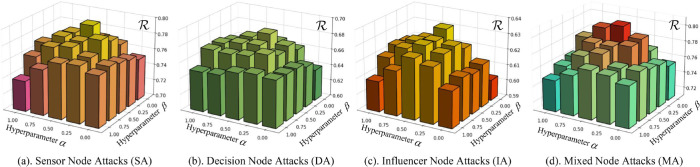
Impact of hyperparameters α and β on resilience ℛ under four attack scenarios: (a) Sensor Node Attacks (SA), (b) Decision Node Attacks (DA), (c) Influencer Node Attacks (IA), and (d) Mixed Node Attacks (MA).

In this subsection, we investigate the influence of the hyperparameters α and β on the resilience performance ℛ of the MPGPD-RC framework. Fig 5 presents a comprehensive 3D surface analysis that evaluates ℛ across four distinct disruption scenarios as a function of the loss weighting coefficients. Each subfigure plots α (the weight assigned to knowledge distillation) along one axis and β (the weight assigned to path-aware contrastive learning) along the other, with the resulting ℛ values rendered on the vertical axis. The color-coded columns correspond to different attack scenarios: Sensor Node Attacks (SA), Decision Node Attacks (DA), Influencer Node Attacks (IA), and Mixed Node Attacks (MA). The results indicate that resilience under DA remains relatively stable across a broad range of (α,β) configurations, suggesting robustness to hyperparameter choice. In contrast, IA and MA exhibit pronounced sensitivity, with sharp fluctuations in ℛ.

Across all four disruption scenarios, the resilience surface consistently peaks at the configuration α=0.5,β=0.25. This setting allocates twice the weight to KL-based decision imitation relative to path-aware contrastive alignment, thereby balancing the gradient contributions of both objectives and preventing the dominance of either component. Under SA and DA conditions, this ratio promotes stable policy convergence by preserving sufficient flexibility in structural modeling without undermining decision fidelity. In the more complex IA and MA settings, where dense inter-node dependencies exacerbate sensitivity to structural perturbations, the chosen configuration effectively mitigates trajectory degradation when β is too low and prevents excessive rigidity in action alignment when β is too high. This parameterization yields a robust equilibrium between structural coherence and behavioral precision. Consequently, α=0.5,β=0.25 is selected as the default setting for all experimental benchmarks.

Further, setting α=0 removes the KL term and yields a PaC–only regime that optimizes exclusively for structural alignment. As shown in Fig 5, this configuration produces a clear reduction in resilience ℛ: without the behavioral anchor provided by ${ℒ}_{KD}$, the student over-emphasizes meta-path regularities and under-utilizes the teacher’s action distribution, which impairs policy quality in scenarios where fine-grained, local corrections are essential to recovery. Conversely, β=0 removes the contrastive term and yields a KD–only regime that imitates the teacher while becoming path-agnostic. Across all attack settings, this variant attains only moderate ℛ because typed, ordered, and directed multi-hop dependencies are not preserved; the policy reproduces marginal action preferences but fails to encode the higher-order semantic routes crucial for coordinated recovery under structural perturbations.

### 5.5 Ablation study

To assess the individual contributions of each component within the MPGPD-RC framework, we conducted an ablation study consisting of three distinct experimental settings. In each setting, a key component of the framework was systematically removed or altered to evaluate its impact on the overall resilience ℛ. The findings of these experiments are summarized in Table 3.

**Table 3:** Ablation study results with 50% attacked nodes.

Model Variant	SA	DA	IA	MA
CGPPO	0.777	0.661	0.611	0.779
MPGPD-RC	**0.798**	**0.684**	**0.635**	**0.813**
w/o MGE	0.771	0.663	0.617	0.789
w/o MPA	0.779	0.669	0.621	0.791

**Without Meta-Path Guided Embeddings (w/o MGE):** In this ablation setting, node representations are derived using a conventional graph convolutional network (GCN) trained in an end-to-end fashion, omitting the meta-path–guided embedding (MGE) module. The MGE component is instrumental in extracting high-fidelity features that encode the structural heterogeneity of the AUS graph, including node topologies, interconnectivity patterns, and higher-order relational semantics.

As shown in Table 3, When the meta-path–guided embedding module is ablated, the representation collapses to a path-agnostic, neighborhood-smoothing regime that largely mirrors the inductive bias of CGPPO, where messages from heterogeneous relations are mixed without preserving typed, ordered, and directed multi-hop semantics; When key nodes are compromised, as in SA or DA scenarios, or when multiple subsystems are simultaneously affected in MA scenarios, the absence of MGE significantly degrades the agent’s ability to discern the altered network structure. This performance deterioration underscores the MGE module’s critical role in enhancing policy robustness by enabling accurate recognition of compromised meta-paths and facilitating structure-aware adaptation during system recovery.

**Without Meta-Path Aware (w/o MPA):** In this ablation setting, the student-side distillation process is entirely omitted. The agent is trained exclusively through the teacher policy using Proximal Policy Optimization (PPO), with no Kullback–Leibler or contrastive alignment mechanisms. The Meta-Path Aware (MPA) module is responsible for integrating global structural semantics by leveraging multi-hop meta-path relationships that reveal latent connections and alternative coordination pathways across the graph.

This capability is especially vital in scenarios involving indirect or distributed attacks, such as IA and MA, where damage propagates through non-adjacent nodes or spans multiple failure zones. The MPA module enables the agent to transcend local neighborhoods, identify structurally meaningful backup routes, and coordinate recovery across dispersed impact regions. By exploiting meta-path semantics, the agent gains a global operational perspective essential for re silient coordination.

Ablation results show a clear decline in resilience performance in the absence of MPA, with notable drops in IA (0.635 vs. 0.621) and MA (0.813 vs. 0.791) scenarios. These findings indicate that without MPA, the agent lacks the ability to leverage global structural cues, limiting its capacity to mitigate widespread or stealthy disruptions. This validates the critical contribution of the MPA module in enhancing resilience by embedding long-range dependencies into the policy learning process.

### 5.6 Convergence analysis

**Fig 6:**
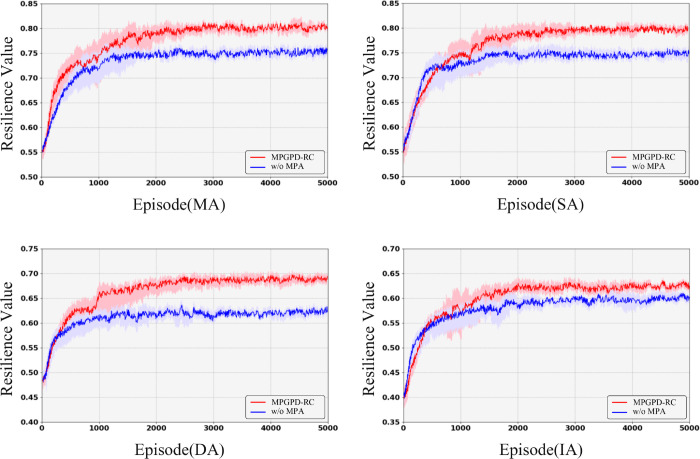
Comparison of resilience value (ℛ) during training for MPGPD-RC (red) and its variant without MPA (blue).

To further evaluate the convergence behavior and assess the impact of Meta-Path guidance on the long-term resilience development, we conducted a longitudinal analysis of system performance across training episodes. As shown in Fig 6, resilience ℛ is tracked under different disruption scenarios, comparing the full MPGPD-RC framework with its Meta-Path Aware–ablated variant (w/o MPA) to highlight the influence of Meta-Path guidance on the convergence of resilience over time.

During the initial 200 training episodes, the configuration without Meta-Path-Aware Policy Distillation (blue curve), which relies solely on the teacher policy with Proximal Policy Optimization (PPO), shows a steeper increase in resilience (ℛ) under Sensor Node Attacks (SA) and Decision Node Attacks (DA). This early advantage arises from the teacher policy’s direct optimization through PPO, which benefits from relaxed meta-path constraints that enable rapid adaptation to the environment. By focusing exclusively on immediate reward signals, the teacher policy achieves high short-term performance quickly, without the added complexity of contrastive alignment or KL divergence distillation, leading to a faster but potentially less robust early convergence.

In contrast, the full MPGPD-RC configuration (red curve), which integrates Meta-Path-Aware Policy Distillation from the beginning, incorporates both KL divergence alignment and path-aware contrastive learning. While this dual-objective approach introduces additional complexity and moderates early convergence, it equips the student policy with a richer understanding of global graph semantics and multi-hop dependencies. This more comprehensive structural awareness slows early learning but strengthens the model’s ability to generalize, reducing the risk of overfitting to local disruptions and fostering more stable long-term convergence.

As training progresses beyond approximately episode 500, the full MPGPD-RC model (red curve) consistently surpasses the teacher-only baseline across all four disruption scenarios—Sensor Node Attacks (SA), Decision Node Attacks (DA), Influencer Node Attacks (IA), and Mixed Node Attacks (MA). By episode 2000, the red curve stabilizes at a resilience value of ℛ≈0.80 under both MA and SA conditions, exhibiting minimal variance. This stabilization reflects the superior long-term convergence of the MPGPD-RC model, with path-aware contrastive loss gradually aligning the student’s trajectory encodings to the global graph structure defined by meta-paths, such as $V_{S}\to V_{D}\to V_{I}\to V_{T}$. This alignment ensures that the model maintains long-range dependencies, leading to improved robustness. At the same time, KL divergence ensures that the student’s learning remains grounded in the teacher’s policy, further enhancing the stability and convergence of the student policy towards optimal behavior.

### 5.7 Time complexity analysis

To assess engineering practicality in terms of convergence efficiency and time cost, we report time-to-convergence and performance under the 50% attack setting in Table 4. Here, **Total time (s)** denotes the total wall-clock training time required to reach a common convergence criterion, **Total episodes** is the number of training episodes to convergence, **per episode (s)** is the mean wall-clock duration per episode (including environment interaction and forward/backward computation), and **Resilience** is the post-convergence performance metric that quantifies coordination under disruptions.

**Table 4:** Time complexity of different methods with 50% attacked nodes (SA).

Model	Total time (s)	Total episodes	per episode (s)	Resilience
CGPPO	5234	3250	1.61	0.777
MPGPD-RC	**4190**	**2354**	**1.78**	**0.798**
w/o MPA	4938	2993	1.65	0.779

Although MPGPD-RC exhibits a slightly longer per-episode duration than CGPPO (1.78 s vs. 1.61 s; +10.6%), it requires substantially fewer episodes to converge (2354 vs. 3250; −27.6%), resulting in a shorter total training time (4190 s vs. 5234 s; −19.9%) and a higher final resilience (0.798 vs. 0.777; +2.7%). The marginal per-episode overhead stems from meta-path–guided representation learning (path-specific attention encoders with contrastive regularization) and teacher–student distillation (trajectory encoding), which introduce constant computational work per episode; however, these structural priors and meta-path masks improve sample efficiency by pruning ineffective exploration and sharpening gradient signals, thereby accelerating convergence despite the slightly longer episode time.

Relative to the full MPGPD-RC, the **w/o MPA** variant exhibits a lower per-episode cost but markedly worse sample efficiency: per-episode time decreases by 7.3% (1.65s vs. 1.78s) owing to the removal of the student branch (GRU trajectory encoding, batch-wise contrastive pairing, and KL/PaC backpropagation), yet the number of episodes to convergence increases by 27.2% (2993 vs. 2354), which translates into a 17.9% higher total time (4938s vs. 4190s) and a lower converged resilience (0.779 vs. 0.798). This trade-off indicates that the meta-path–aware distillation in MPGPD-RC, despite incurring a modest per-episode overhead, substantially improves sample efficiency by anchoring the student’s action distribution (KL) and preserving trajectory-level meta-path semantics (PaC), thereby reducing the number of required learning episodes and yielding higher final robustness.

## 6 Conclusion

This work presented **MPGPD-RC**, a meta-path–guided policy distillation framework for resilient coordination in Autonomous Unmanned Swarms (AUS). The approach couples (i) a meta-path–guided embedding module that encodes typed, ordered, and directed multi-hop relations into semantically coherent node representations, with (ii) a meta-path–aware distillation module that transfers a PPO teacher’s behavior to a lightweight student by jointly aligning action distributions (KL) and trajectory-level structural codes (path-aware contrastive learning). By injecting domain meta-path semantics and employing relaxed masking for exploration, MPGPD-RC integrates local and global relational cues into the decision pipeline, yielding policies that remain effective under severe, structured disruptions. Across sensor-, decision-, influencer-, and mixed-attack scenarios and multiple intensities, the framework consistently surpasses competitive baselines and ablations, demonstrating that preserving meta-path semantics materially improves both robustness and coordination quality. In future work, we will explore adaptive meta-path selection to develop a unified resilience-enhancement architecture transferable across various AUS topologies and tasks. Additionally, we aim to establish industry-standard resilience benchmarks, as there is currently a lack of standardized metrics and data for real-world environments, and evaluate the scalability and real-time feasibility of our approach through cross-validation in physical settings.
